# Transcriptome profiling of the spermatheca identifies genes potentially involved in the long-term sperm storage of ant queens

**DOI:** 10.1038/s41598-017-05818-8

**Published:** 2017-07-20

**Authors:** Ayako Gotoh, Shuji Shigenobu, Katsushi Yamaguchi, Satoru Kobayashi, Fuminori Ito, Kazuki Tsuji

**Affiliations:** 1 Department of Biology, Faculty of Science and Engineering and Institute for Integrative Neurobiology, Konan University, 8-9-1 Okamoto, Higashinada-ku, Kobe 658-8501 Japan; 2 NIBB Core Research Facilities, National Institute for Basic Biology, Okazaki, 444-8585 Japan; 3 Department of Basic Biology, Faculty of Life Science, SOKENDAI (The Graduate University for Advanced Studies), Okazaki, Aichi 444-8585 Japan; 4 Life Science Center of Tsukuba Advanced Research Alliance (TARA Center), University of Tsukuba, Tsukuba, Ibaraki 305-8577 Japan; 5 Faculty of Agriculture, Kagawa University, Ikenobe, Miki 761-0795 Japan; 6 Department of Agro-Environmental Sciences, Faculty of Agriculture, University of the Ryukyus, Nishihara, Okinawa 903-0213 Japan

## Abstract

Females of social Hymenoptera only mate at the beginning of their adult lives and produce offspring until their death. In most ant species, queens live for over a decade, indicating that ant queens can store large numbers of spermatozoa throughout their long lives. To reveal the prolonged sperm storage mechanisms, we identified enriched genes in the sperm-storage organ (spermatheca) relative to those in body samples in *Crematogaster osakensis* queens using the RNA-sequencing method. The genes encoding antioxidant enzymes, proteases, and extracellular matrix-related genes, and novel genes that have no similar sequences in the public databases were identified. We also performed differential expression analyses between the virgin and mated spermathecae or between the spermathecae at 1-week and 1-year after mating, to identify genes altered by the mating status or by the sperm storage period, respectively. Gene Ontology enrichment analyses suggested that antioxidant function is enhanced in the spermatheca at 1-week after mating compared with the virgin spermatheca and the spermatheca at 1-year after mating. *In situ* hybridization analyses of 128 selected contigs revealed that 12 contigs were particular to the spermatheca. These genes have never been reported in the reproductive organs of insect females, suggesting specialized roles in ant spermatheca.

## Introduction

Reproductive success is crucial for sexual organisms, and a great diversity of reproductive strategies have been evolved in each species and sex, including copulation behaviours, sperm competition, investment for gamete production, efficiency of fertilization, and parental care. Female sperm storage from mating to fertilization is a major reproductive strategy and is associated with reproductive life cycles and post-copulatory sexual selection among most insects and some vertebrates^1^. In social Hymenoptera such as ants, social wasps, and bees, females have prominent long-term sperm storage abilities according to their specialized life history. Reproductive females (queens) only mate at the beginning of their adult lives and subsequently maintain viable sperms in their spermatheca until their death. Lifespans of social hymenopteran queens are relatively longer than those of other insects; honeybee queens usually live for 2–4 years^2^, ant queens of most species can live for more than 10 years and some for several decades^3^. Moreover, a large amount of stored sperm is necessary for keeping their large colony (e.g. honeybees produce more than 1 million offspring^4^ and several million offspring are born from queens of army ants and leaf-cutting ants^5^), and queens and their colony members increase reproductive fitness when queens maintain large numbers of sperm for long periods because sexual castes of subsequent generations are usually produced after colony growth, which may take several years. Furthermore, evolution of the prominent sperm storage ability is also provide crucial insight into the transitional process from primitive to advanced eusociality in Hymenoptera because reproductive females of hymenopteran species with advanced eusociality tend to have traits of longer longevity and more colony member production than those with primitive eusociality^5, 6^.

Components of spermatheca fluids from honeybee queens and secretions from male bee accessory glands have been investigated for the past 40–50 years in efforts to reveal long-term sperm storage mechanisms. Accordingly, multiple candidate factors for sperm longevity have been considered, including ions, sugars, pH, and enzymes of antioxidant and energy metabolism^7–15^. However, it remains unclear whether these candidates are truly important for prolonged sperm storage in honeybees. Ants evolved the ability of queens to store sperm for an extremely longer time than honeybees (see above), therefore they are also useful for studies of the prolonged sperm storage mechanisms. Furthermore, they also shed light on evolutionary history of the sperm storage systems in social Hymenoptera because ants and honeybees independently evolved advanced eusociality. However there is a few studies of sperm maintenance in ants^16^. Because spermatheca directly influence sperm conditions, we investigated spermatheca functions in ant queens as the first step to reveal details of the ensuing long-term sperm storage mechanisms.

Morphological traits of female sperm storage organs are highly diverse among insects and have been closely associated with sperm competition, sexual conflict, and storage function^17–19^. In ant queens, the spermatheca comprises a spermathecal reservoir, a pair of spermathecal glands, a spermatheca duct connecting the reservoir and common oviduct, and a sperm pump^20–22^. The spermathecal reservoir wall comprises two simple epithelial cell types with a cuticle lining, columnar epithelia in the hilar region of the reservoir near the opening of the spermathecal duct, and squamous epithelia in the distal region. Ultrastructural observations indicate that the columnar cells of these reservoir walls are abundant in mitochondria and apical microvilli, indicating active transporting functions. However, the squamous epithelial cells contain few mitochondria and lack microvilli, suggesting no cellular activities^20–22^. Moreover, both cell types have poorly developed endoplasmic reticulum and golgi apparatus, suggesting the absence of secretory functions. The structure of the reservoir wall of ant queens is unique among social hymenopteran species because in social bees and wasps, this reservoir wall has uniform thickness and comprises the columnar epithelial cells^6, 23–28^. The spermathecal gland contains glandular and central duct cells, which were classified as type-3 secretory cells^29^. In later studies, secretions from these cells reportedly affected sperm viability in honeybee queens^30^. The sperm pump comprises muscular layers that are located at the distal portion of the spermathecal duct and may regulate sperm migration into the spermathecal reservoir after mating or sperm release prior to insemination. Although these morphologies are well characterized, little is known of the molecular functions of the spermatheca in ant queens.

**Figure 1:**
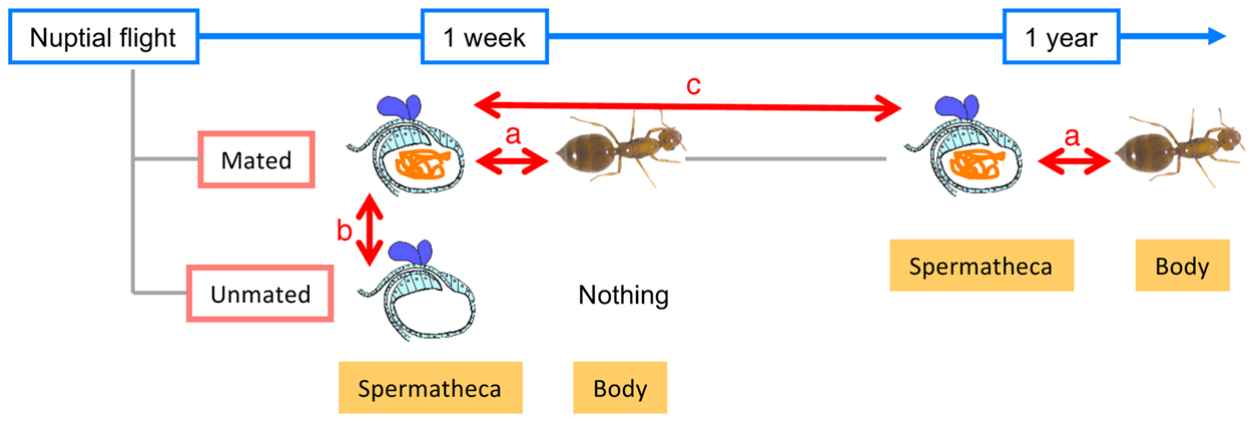
Sample preparation schematic: Differential gene expressions patterns were compared between (**a**) the spermatheca and the body, (**b**) the spermatheca from virgin and inseminated queens, and (**c**) the spermatheca at 1 week and 1 year after mating.

Proteome analyses have been performed in the spermatheca from virgin and inseminated queen honeybees and in *Atta sexdens rubropilosa* ants^12, 31^. However, spermatheca-specific functions are poorly elucidated from the protein expression studies of the spermatheca because the ensuing protein profiles may represent housekeeping proteins that are also abundantly expressed in other tissues, warranting comparative studies of the spermatheca and other tissues. RNA sequencing methods using next generation high-throughput sequencing technologies can be used to determine large-scale gene expression profiles even in non-model organisms. Hence, in the present study, we screened candidate genes that contribute to sperm storage functions using various differential gene expression analyses of the spermatheca and the body samples from *Crematogaster osakensis* ant queens using the RNA sequencing method (Fig. 1). Several thousand *C*. *osakensis* queens can be collected immediately after nuptial flight. They can be easily kept in the laboratory and can often be maintained for over 7 years^32^, offering a highly convenient model for studies of sperm storage. Firstly, we identified genes enriched in the spermatheca compared with those in the body samples to characterize the spermatheca functions (a in Fig. 1). In insect females, behavioural and physiological changes have been characterized following sperm and seminal fluid transfer^33–35^. Furthermore, gene expression profiles change in accordance with the mating status in the bodies and reproductive tracts, and these changes are considered important for adaptive functions, such as sperm competition and sperm storage, relating to reproductive success^35–41^. Therefore, we secondarily investigated differentially expressed spermatheca genes before and after mating to detect enhanced genes in the spermatheca during sperm maintenance compared with those in the spermatheca without spermatozoa (b in Fig. 1). Third, we also analysed gene expression changes in the spermatheca at 1 week and 1 year after mating because we expected that genes with enhanced expression in later stage after sperm storage are likely involved in long-term sperm storage mechanisms compared with those in initial stage (c in Fig. 1).

To the best of our knowledge, this is the first molecular study of the spermatheca in ants, and the present *de novo* sequencing analyses identified multiple candidate genes for the long-term sperm storage. Finally, *in situ* hybridization analyses confirmed the spatial expression patterns of selected genes, resulting in the discovery of 12 spermatheca specific contigs.

## Results

### RNA-sequencing and assembly

To build a comprehensive gene catalogue, we constructed RNA-seq libraries from the bodies of mated queens at 1 week and 1 year after mating, workers and males, and spermathecae of virgin and mated queens at 1 week and those of mated queens at 1year after nuptial flight and accessory glands of males (Supplementary Table S1). Paired-end sequencing of the 23 cDNA libraries from the samples using the Illumina Hiseq 2000 platform yielded a total of 384.4 million reads. We assembled the RNA-seq reads de novo by Trinity program^42^ (v. r2012-06-08) resulting in 164,805 contigs with a mean length of 2008.5 bp ranging from 201 to 75,590 bp. We predicted 80,424 ORFs from the assembly, among which 37,870 (47.1%) matched to the proteins in the NCBI nr protein database (BLASTP, cutoff e-value of 1.0e-4), and 21,833 and 14,894 were matched with Arthropoda (57.7%) and bacteria sequences (39.3%) in top hits. After removing the 14,894 ORFs with hits to bacterial sequences, assuming them as bacterial contaminations, and 12,005 ORFs with extremely low expression (RPKM values in all 23 samples of less than 1), the remaining 53,525 ORFs with a N50 of 1230 bp were defined as a reference gene set of *C*. *osakensis*. To evaluate the accuracy of the assembly and to prepare *in situ* hybridization probes, we cloned 128 contigs and subjected them to Sanger sequencing. These sequences were more than 98% identical to those generated by our *de novo* assembly, indicating a successful *de novo* assembly.

### Genes enriched in the spermatheca

**Figure 2:**
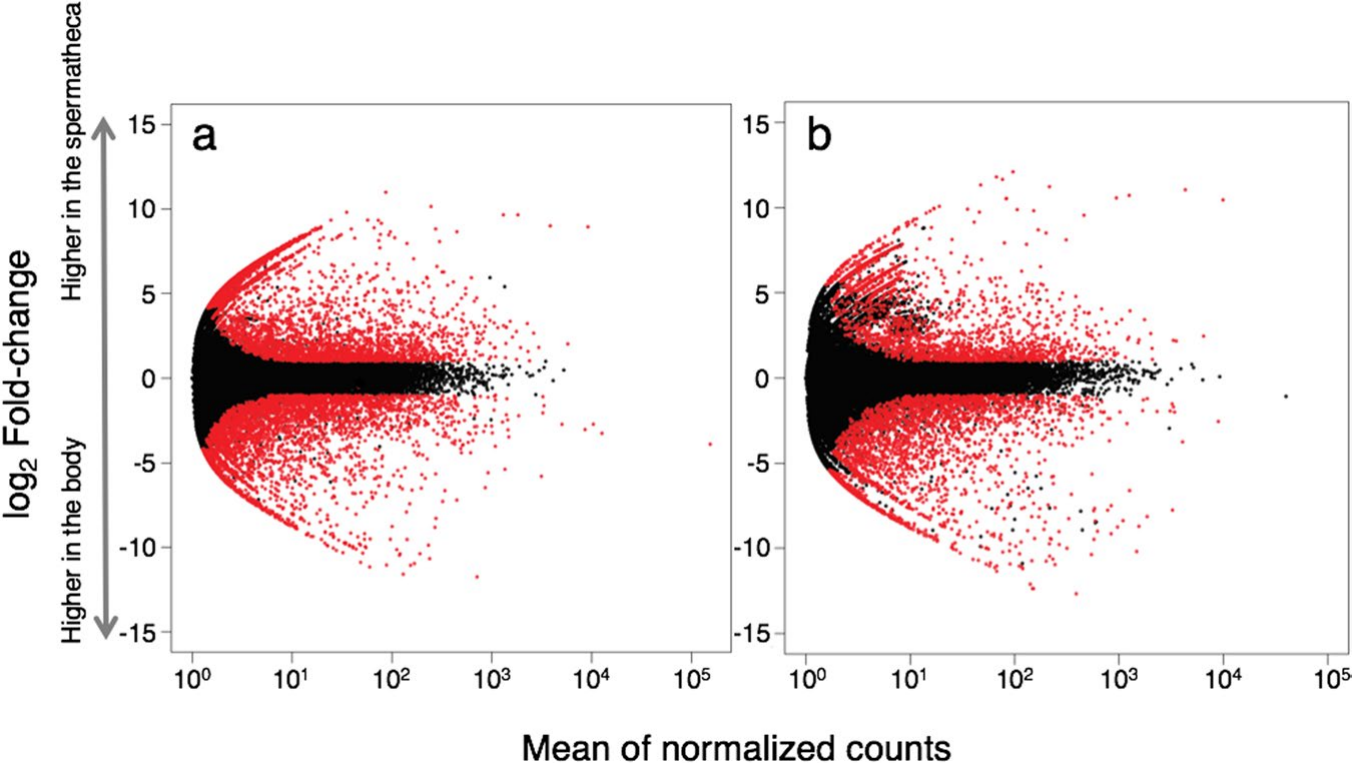
Plots of differentially expressed genes between the spermatheca and the body samples at 1 week (**a**) and 1 year (**b**) after mating. Red dots indicate differentially expressed genes with false discovery rates (FDR) of <0.01and |log_2_ fold change| ≥ 1.

We conducted differential expression analyses of the spermatheca and their body samples after 1 week and 1 year of mating (a in Fig. 1) with three biological replicates for each tissue using DEseq2^43^. In these analyses, 5,941 and 2,794 genes were up-regulated and 3,785 and 2,741 genes were down-regulated in the spermatheca samples compared with those in the body samples after 1 week and 1 year of mating, respectively, with false discovery rate (FDR) < 0.01 and |log_2_ fold change| ≥ 1 (Fig. 2a,b). Among them, 2,477 spermatheca-enriched genes were common to both comparisons after 1 week and 1 year of mating. These overrepresented genes showed a wide variety of function, such as antioxidant enzymes, chaperones, and energy metabolism enzymes as well as novel genes that have no similar sequences in the public database (Table 1).

**Table 1:** List of selected genes expressed in the spermatheca.

Category	Contig No.	Predicted genes	Predicted protein motif	Signal sequence	Fold changes (Numbers in the bracket are not significant at 0.01)				Figures of *in situ* hybridization	Expression pattern^‡^
					Spermatheca/body		Spermatheca/spermatheca			
					1 week	1 year	mated/unmated	1 year/1 week		
Antioxidant	comp70976_c0_seq1.m.342323	PREDICTED: uncharacterized protein LOC105565138	Animal haem peroxidase	—	6.28	5.08	0.48	−1.24	—*	Failed
“	comp56723_c0_seq1.m.35720	superoxide dismutase	Iron/manganese superoxide dismutases, C-terminal domain	−	1.05	1.32	−0.31	(−0.05)	—*	HE, MG, OV
“	comp72365_c0_seq1.m.514034	glutaredoxin-c4-like isoform	Glutaredoxin	—	1.04	1.36	(−0.28)	(0.09)	−*	GC, HE, MG, OV, SD, SG
“	comp71714_c0_seq12.m.455490	glutathione s-transferase isoform c-like isoform	Glutathione S-transferase, N-terminal domain	—	(0.57)	1.98	−0.36	(0.31)	Fig. 5b	MG, SG
“	comp33014_c0_seq1.m.14779	peroxiredoxin- mitochondrial	Redoxin	—	(0.49)	1.57	−0.42	(0.25)	Fig. 5c	MG, SG
Chaperone	comp63273_c0_seq1.m.58938	protein lethal essential for life-like	Hsp20/alpha crystallin family	—	2.89	2.83	−0.43	0.94	Fig. 5d	OV, SG
“	comp71039_c10_seq1.m.351826	protein charybde-like	RTP801 C-terminal region	—	2.60	2.67	(−0.47)	(−0.42)	Fig. 5e	HE, MG, OV, SG
“	comp69851_c2_seq1.m.223848	heat shock 70 kda protein cognate 4	Hsp70 protein	—	2.34	1.67	(0.03)	(−0.22)	Fig. 5f	HE, MG, OV, SG
“	comp70616_c1_seq1.m.300138	dnaj homolog subfamily c member 22	DnaJ domain	—	1.87	2.01	(−0.47)	(0.70)	—*	OV
“	comp71512_c0_seq2.m.419572	heat shock protein ddb_g0288861-like isoform	—	—	1.16	1.41	(0.21)	0.63	—	—
Transporter and channel	comp66765_c0_seq1.m.99406	nose resistant to fluoxetine protein 6	Acyltransferase family	—	8.47	6.96	(0.36)	−0.99	—*	Failed
“	comp70810_c0_seq1.m.322233	sodium-independent sulfate anion transporter	STAS domain	—	5.98	4.64	(0.18)	(0.14)	—*	HE, MG, OV, SG
“	comp66171_c0_seq1.m.88333	low quality protein: bestrophin-3-like	Bestrophin, RFP-TM, chloride channel	—	5.08	5.46	(0.19)	(0.01)	—*	HE, MG, OV, SG
“	comp70257_c3_seq1.m.260125	band 3 anion transport protein isoform	HCO3- transporter family	—	4.75	3.84	(0.41)	(−0.54)	Fig. 5g	HE, MG, OV
“	comp69671_c1_seq1.m.212720	facilitated trehalose transporter tret1	Sugar (and other) transporter	—	4.59	4.36	(−0.04)	(0.26)	—*	MG, OV, SG
“	comp71363_c1_seq23.m.401544	ammonium transporter rh type a isoform	Ammonium Transporter Family	—	4.24	4.70	(−0.62)	1.67	Fig. 6a	SG
“	comp70992_c5_seq1.m.344700	facilitated trehalose transporter tret1-like	Sugar (and other) transporter	—	3.72	3.68	(−0.08)	(0.05)	Fig. 5h	MG, OV, SG
“	comp69444_c0_seq1.m.194515	multidrug resistance-associated protein 4-like	ABC transporter	—	3.35	3.31	−0.61	(0.45)	Fig. 6b	SG
“	comp67902_c1_seq1.m.126719	proton-coupled amino acid transporter 1-like isoform	Transmembrane amino acid transporter protein	—	3.23	3.58	(−0.14)	(−0.08)	Fig. 6c	SG
“	comp70337_c0_seq1.m.269473	potassium voltage-gated channel subfamily h member 2 isoform	Ion transport protein	—	1.73	2.60	−0.55	(−0.11)	Fig. 5i	HE, MG, OV, SG (central duct)
Energy metabolism	comp70771_c0_seq1.m.316801	maltase a2-like isoform	Alpha amylase, catalytic domain	—	4.35	3.13	(0.04)	−0.49	—	—
“	comp55829_c0_seq1.m.33328	l-lactate dehydrogenase-like	lactate/malate dehydrogenase, NAD binding domain	—	3.75	3.90	(0.01)	(−0.10)	—	—
“	comp67380_c0_seq1.m.112336	hexokinase type 2 isoform	Hexokinase	—	3.30	4.06	(−0.05)	(0.11)	Fig. 5j	MG, OV, SG
“	comp71645_c0_seq1.m.442441	glucose dehydrogenase	GMC oxidoreductase	—	3.01	3.43	(−0.04)	(0.29)	—*	MG, SG
“	comp71373_c0_seq1.m.402567	succinate dehydrogenase	FAD binding domain	—	2.95	2.68	(−0.06)	(−0.30)	—	—
Extracellular matrix related	comp63322_c0_seq1.m.59242	flocculation protein flo11-like	Chitin binding Peritrophin-A domain	—	8.66	9.56	0.73	(0.00)	Fig. 6d	SG
“	comp64346_c0_seq1.m.68928	flocculation protein flo11-like	Chitin binding Peritrophin-A domain	YES	9.16	10.55	0.78	(0.04)	—*	MG, OV, SG
“	comp63194_c1_seq1.m.58387	chondroitin proteoglycan-2-like	Chitin binding Peritrophin-A domain	YES	6.72	5.06	(−0.22)	−0.94	—*	HE, MG, OV, SG
“	comp71322_c9_seq1.m.393320	peroxidasin	—	—	6.64	7.35	(0.12)	(−0.15)	Fig. 6e	SG
“	comp68902_c1_seq1.m.163530	dentin matrix protein 4-like protein	—	—	5.43	3.89	(−0.14)	(−0.57)	Fig. 6f	SG
“	comp70138_c0_seq1.m.249533	hyaluronidase-like	Hyaluronidase	YES	3.95	3.84	−0.72	−0.77	—	—
“	comp63113_c0_seq1.m.57806	extracellular matrix protein 2 isoform	Leucine rich repeat	—	1.81	1.09	(0.00)	(−0.21)	—*	MG, OV, SG
“	comp61774_c0_seq1.m.51004	procollagen-lysine,2-oxoglutarate 5-dioxygenase 3	—	YES	(0.10)	1.04	(−0.11)	0.83	—	—
Protease	comp65714_c0_seq1.m.81458	trypsin epsilon-like	Trypsin	—	9.66	10.58	(−0.36)	−0.51	—	—
“	comp56664_c0_seq1.m.35571	a disintegrin and metalloproteinase with thrombospondin motifs	Reprolysin family propeptide	YES	4.73	3.62	(0.26)	(−0.17)	—*	Failed
“	comp71039_c3_seq1.m.351761	thyrotropin-releasing hormone-degrading ectoenzyme-like	ERAP1-like C-terminal domain	—	4.55	4.20	(−0.04)	−0.75	—*	CO, GC, SG
“	comp65462_c0_seq1.m.78928	angiotensin-converting enzyme-like	Angiotensin-converting enzyme	YES	4.25	2.77	(0.15)	−1.13	Fig. 5k	OV, SG
Protease inhibitor	comp70125_c3_seq13.m.248874	plasminogen activator inhibitor 1	Serpin (serine protease inhibitor)	YES	3.42	3.95	(0.07)	0.38	Fig. 5l	OV, SG
Others	comp56610_c0_seq1.m.35452	No hits	—	—	11.00	11.81	(−0.41)	(−0.42)	Fig. 5m	OV, SG
“	comp65548_c0_seq1.m.79879	PREDICTED: uncharacterized protein LOC105561087	Zona pellucida-like domain	YES	8.83	8.52	(0.21)	(−0.48)	Fig. 6k	HE
“	comp63251_c0_seq1.m.58816	nicotinamidase-like	—	—	8.66	7.12	(0.27)	−1.52	Fig. 6g	SG (central duct)
“	comp65667_c0_seq1.m.81045	No hits	—	—	8.31	9.89	1.02	(−0.07)	—	—
“	comp71576_c7_seq1.m.430348	No hits	—	—	6.84	7.80	(1.66)	(−0.18)	Fig. 6j	HE
“	comp70080_c1_seq1.m.244755	secreted beta-glucosidase adg3 isoform x1	—	—	6.50	(−2.99)	1.44	−6.13	—	—
“	comp62775_c0_seq1.m.55873	protein lethal malignant blood neoplasm 1	Insect cuticle protein	YES	6.06	8.70	(0.59)	1.76	—*	OV, SG
“	comp68918_c0_seq1.m.164810	prostatic acid phosphatase-like	Histidine phosphatase superfamily (branch 2)	YES	5.69	5.11	(−0.33)	−1.34	—	—
“	comp71109_c1_seq1.m.360019	protein lozenge	Runt domain	—	5.64	5.54	(0.27)	−0.65	Fig. 5n	MG, OV, SG (central duct cell)
“	comp71230_c1_seq1.m.377439	No hits	—	YES	5.27	4.35	(0.08)	(−0.13)	Fig. 6l	HE, SG (central duct cell)
“	comp70822_c1_seq1.m.323463	ets translocation variant 1	Ets-domain	—	5.09	5.88	1.05	(0.28)	—*	FB, GC, HE, MG, OV, SD, SG
“	comp70117_c0_seq1.m.248081	probable gpi-anchored adhesin-like protein pga55	Chitin binding Peritrophin-A domain	YES	3.61	2.88	−1.58	(−0.30)	—	—
“	comp55892_c0_seq1.m.33472	protein takeout-like	Haemolymph juvenile hormone binding protein (JHBP)	YES	3.47	2.24	−1.02	−1.12	—*	MG, OV
“	comp65714_c0_seq1.m.81456	protein unc-13 homolog d isoform	C2 domain	—	2.66	2.57	(−0.01)	(0.33)	Fig. 6h	SG
“	comp68745_c0_seq1.m.156391	xanthine dehydrogenase	Molybdopterin-binding domain of aldehyde dehydrogenase	—	2.04	2.18	(−0.22)	(0.70)	Fig. 6i	SG
“	comp62522_c0_seq1.m.54679	lebercilin-like protein isoform x1	Ciliary protein causing Leber congenital amaurosis disease	—	1.92	(−0.09)	2.35	−1.66	—	—
“	comp70881_c2_seq1.m.331137	nuclear hormone receptor ftz-f1 beta isoform x1	Ligand-binding domain of nuclear hormone receptor	—	1.25	(0.49)	(0.01)	(−0.30)	—	—
“	comp72284_c0_seq1.m.514027	pheromone-binding protein gp-9-like	PBP/GOBP family	YES	1.19	1.46	0.83	3.37	Fig. 5o	GC, SG
“	comp65703_c0_seq1.m.81364	vitellogenin precursor	Lipoprotein amino terminal region	YES	−3.90	(−1.08)	4.17	(−0.08)	—	—
*Figures are deposited in figshare (http://dx.doi.org/10.6084/m9.figshare.4750072)										

^‡^Failed = no signals, GC = genital chamber, SG = spermathecal gland, HE = hilar columnar epithelial cells of spermatheca reservoir, SD = spermathecal duct, OV = ovary, MG = midgut, CO = common oviduct.

Gene Ontology (GO) enrichment analyses of genes with differential expression levels in the spermatheca were performed using Fisher’s exact test. GO terms associated with transmembrane transporters (GO:0022857, GO:0055085) and oxidoreductase activity (GO:0016491) were significantly enriched in both up- and down-regulated genes of the spermatheca compared with the body samples after 1 week and 1 year of mating, relative to all annotated genes (Table 2). GO terms for precursor metabolites and energy (biological process, GO:0006091), ATPase activity (molecular function, GO:0016887), and mitochondrial function (cellular components, GO:0005739) were enriched only among genes that were highly expressed in the spermatheca (Table 2).

**Table 2:** Over-represented gene ontology terms among genes differentially expressed between the spermatheca and the body samples of queens after 1 week and 1 year of mating.

Category	Term	GO-ID	FDR
Up-regulated genes in the spermatheca at 1 week after mating			
MF	transmembrane transporter activity	GO:0022857	2.36E-15
BP	generation of precursor metabolites and energy	GO:0006091	2.13E-07
BP	small molecule metabolic process	GO:0044281	1.55E-06
MF	oxidoreductase activity	GO:0016491	2.94E-06
MF	ATPase activity	GO:0016887	1.34E-04
CC	extracellular region part	GO:0044421	8.49E-03
CC	mitochondrion	GO:0005739	8.97E-03
CC	vacuole	GO:0005773	8.97E-03
Down-regulated genes in the spermatheca at 1 week after mating			
MF	oxidoreductase activity	GO:0016491	3.13E-15
MF	hydrolase activity, acting on carbon-nitrogen (but not peptide) bonds	GO:0016810	1.82E-03
MF	peptidase activity	GO:0008233	1.82E-03
BP	transmembrane transport	GO:0055085	1.50E-02
CC	extracellular space	GO:0005615	1.82E-02
MF	lyase activity	GO:0016829	1.94E-02
BP	lipid metabolic process	GO:0006629	2.74E-02
BP	small molecule metabolic process	GO:0044281	2.86E-02
Up-regulated genes in the spermatheca at 1 year after mating			
MF	transmembrane transporter activity	GO:0022857	1.23E-16
MF	oxidoreductase activity	GO:0016491	4.37E-15
BP	generation of precursor metabolites and energy	GO:0006091	2.00E-08
CC	mitochondrion	GO:0005739	2.50E-08
BP	small molecule metabolic process	GO:0044281	1.15E-06
CC	vacuole	GO:0005773	2.91E-03
MF	ATPase activity	GO:0016887	6.34E-03
CC	extracellular region	GO:0005576	7.69E-03
Down-regulated genes in the spermatheca at 1 year after mating			
MF	oxidoreductase activity	GO:0016491	4.01E-11
MF	transmembrane transporter activity	GO:0022857	2.40E-04
BP	transmembrane transport	GO:0055085	2.40E-04
CC	extracellular space	GO:0005615	3.49E-02
MF	hydrolase activity, acting on carbon-nitrogen (but not peptide) bonds	GO:0016810	4.71E-02

Abbreviations: BP, biological process; MF, molecular function; CC, cellular components.

### Differentially expressed genes between the spermathecae with and without sperm

**Figure 3:**
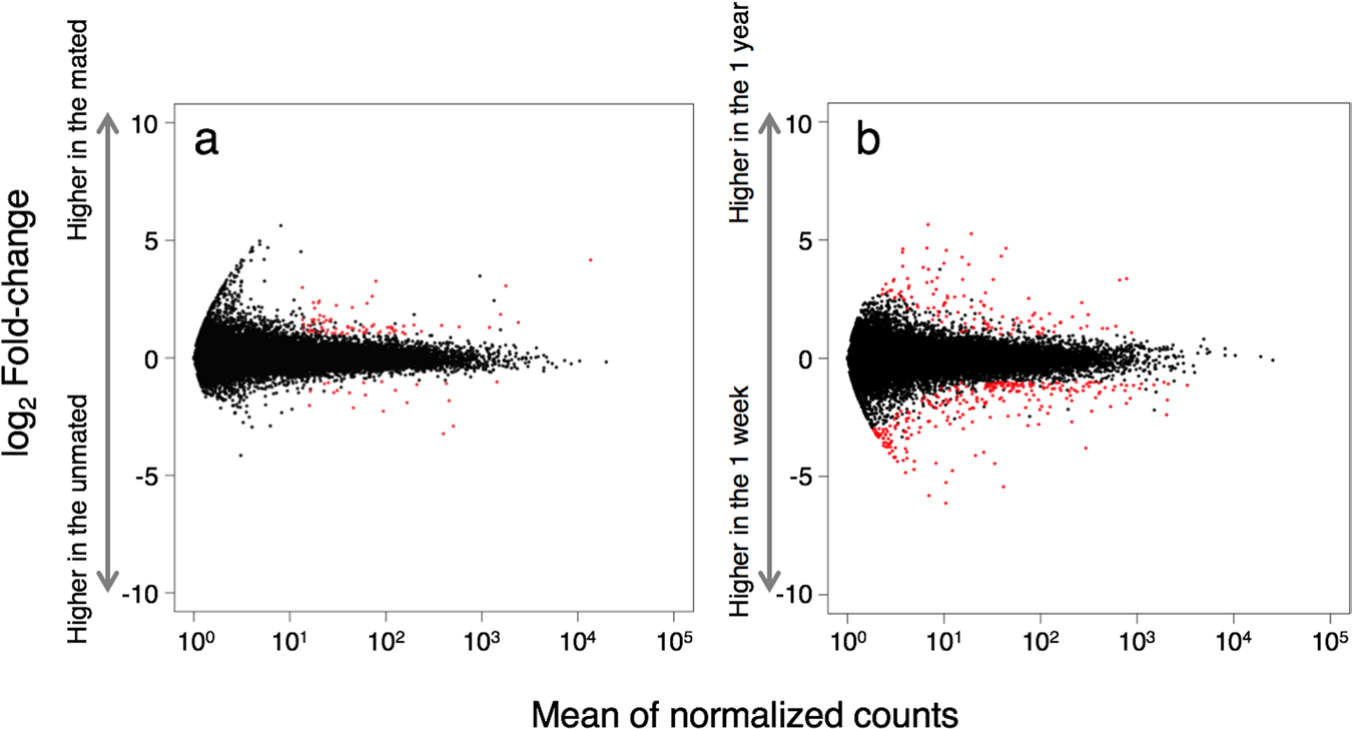
Plots of differentially expressed genes between the spermathecae of inseminated vs. virgin ant queens (**a**) and between the spermathecae at 1 week and 1 year after mating (**b**). Red dots indicate differentially expressed genes (FDR < 0.01 and |log_2_ fold change| ≥ 1).

To determine genes triggered by the start of sperm storage in the spermatheca, we investigated the transcriptomic change before and after mating in the spermatheca (b in Fig. 1). Our RNA-seq analysis revealed that 75 genes were up-regulated and 20 genes were down-regulated in the spermatheca of inseminated queens compared with those in the spermatheca of virgin queens (FDR < 0.01 and |log_2_ fold change| ≥ 1, Fig. 3a).

Among the up-regulated 75 genes induced by sperm storage, genes annotated as oxidoreductase activity (GO:0016491) were significantly enriched compared with all annotated genes from all samples (FDR < 0.05).

**Figure 4:**
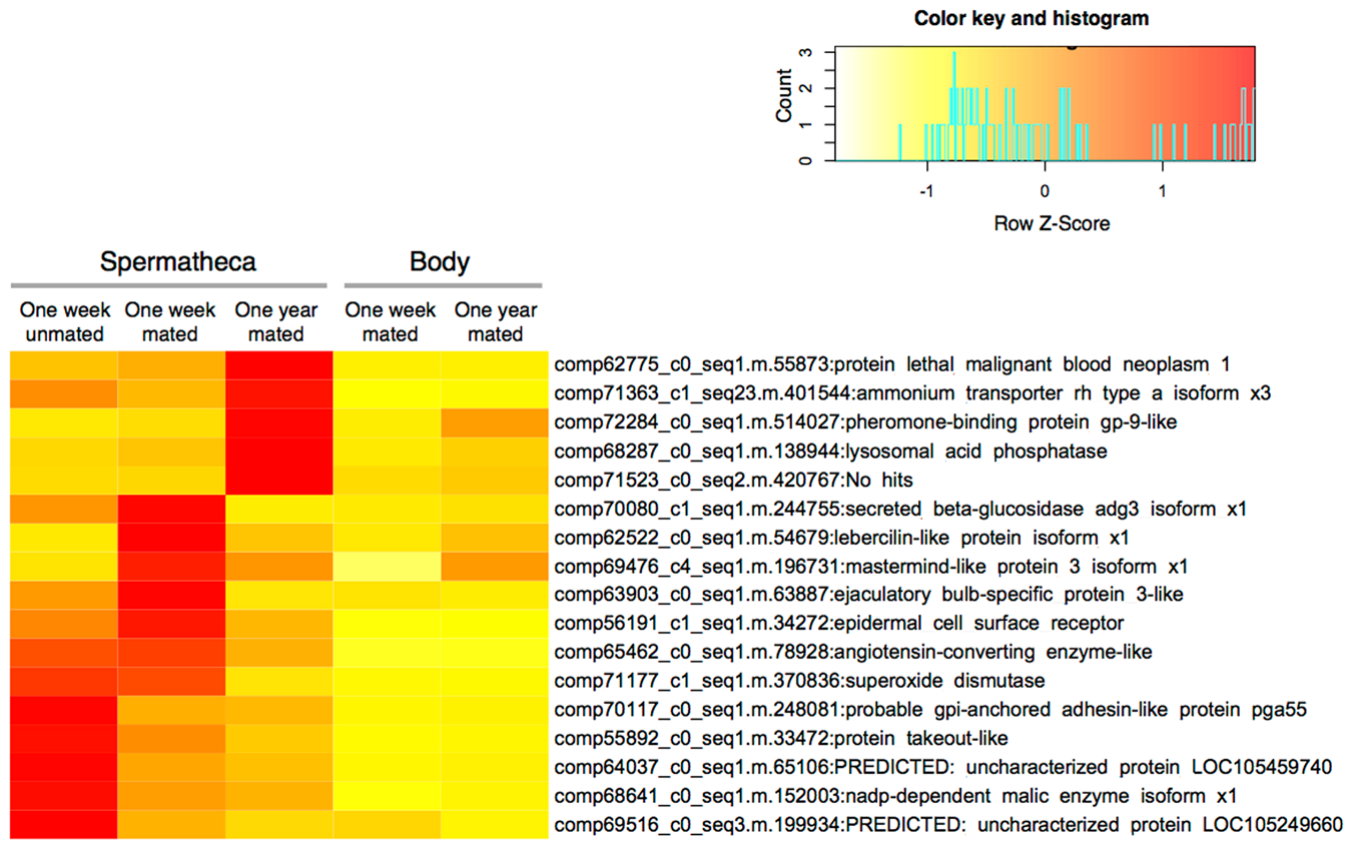
Heat map of selected genes that were regulated by mating status or sperm storage period in the spermathecae. Reads per million (RPM) values of the spermatheca and the body samples were calibrated to Z-scores.

Of the 75 up-regulated genes in the spermatheca with spermatozoa, 11 genes (for instance, comp65667_c0_seq1.m.81045: No hits and comp70822_c1_seq1.m.323463: ETS translocation variant 1), were also enriched in the spermatheca compared with those in the body samples at 1 week after mating (Table 1 and Fig. 4). Genes, such as probable GPI-anchored adhesin-like protein pga55 (comp70117_c0_seq1.m.248081) and protein takeout-like (comp55892_c0_seq1.m.33472), were abundant in the spermatheca of virgin queens compared with the spermatheca and the body samples of mated queens (Table 1 and Fig. 4).

### Gene expression changes with the duration of sperm storage

To identify up-regulated genes during long-term sperm storage, we compared the spermatheca at 1 week and at 1 year after mating (c in Fig. 1). Accordingly, the analyses of differentially expressed genes identified 112 and 264 genes that were increased and decreased, respectively, in the spermatheca at 1 year compared with those at 1 week after mating (FDR < 0.01 and |log_2_ fold change| ≥ 1, Fig. 3b).

GO enrichment analysis revealed that genes related oxidoreductase activity (GO:0016491) were significantly overrepresented in the highly-expressed genes of the spermatheca at 1 weeks than at 1 years after mating compared with all annotated genes (FDR < 0.005).

Among the 112 up-regulated genes in the spermatheca at 1 year after mating, 24 genes, such as those encoding pheromone-binding protein gp-9-like (comp72284_c0_seq1.m.514027), protein lethal malignant blood neoplasm 1 (comp62775_c0_seq1.m.55873), and ammonium transporter rh type A isoform (comp71363_c1_seq23.m.401544), were also enriched in the spermatheca after 1 year of mating compared with those in the body samples (Table 1 and Fig. 4).

### Spatial gene expression patterns

To determine spatial expression patterns of candidate genes for long–term sperm storage mechanisms in various spermatheca parts, we investigated localizations of 128 contigs in the abdomens of ant queens using *in situ* hybridization. The 128 contigs were preferentially selected from the list of differentially expressed genes, such as antioxidant enzymes, chaperones, transporters, and genes with large numbers of reads and high log fold changes in the spermatheca relative to the body samples, and contigs that were altered by mating status or sperm storage periods (Table 1 and Supplementary Dataset 1).

**Figure 5:**
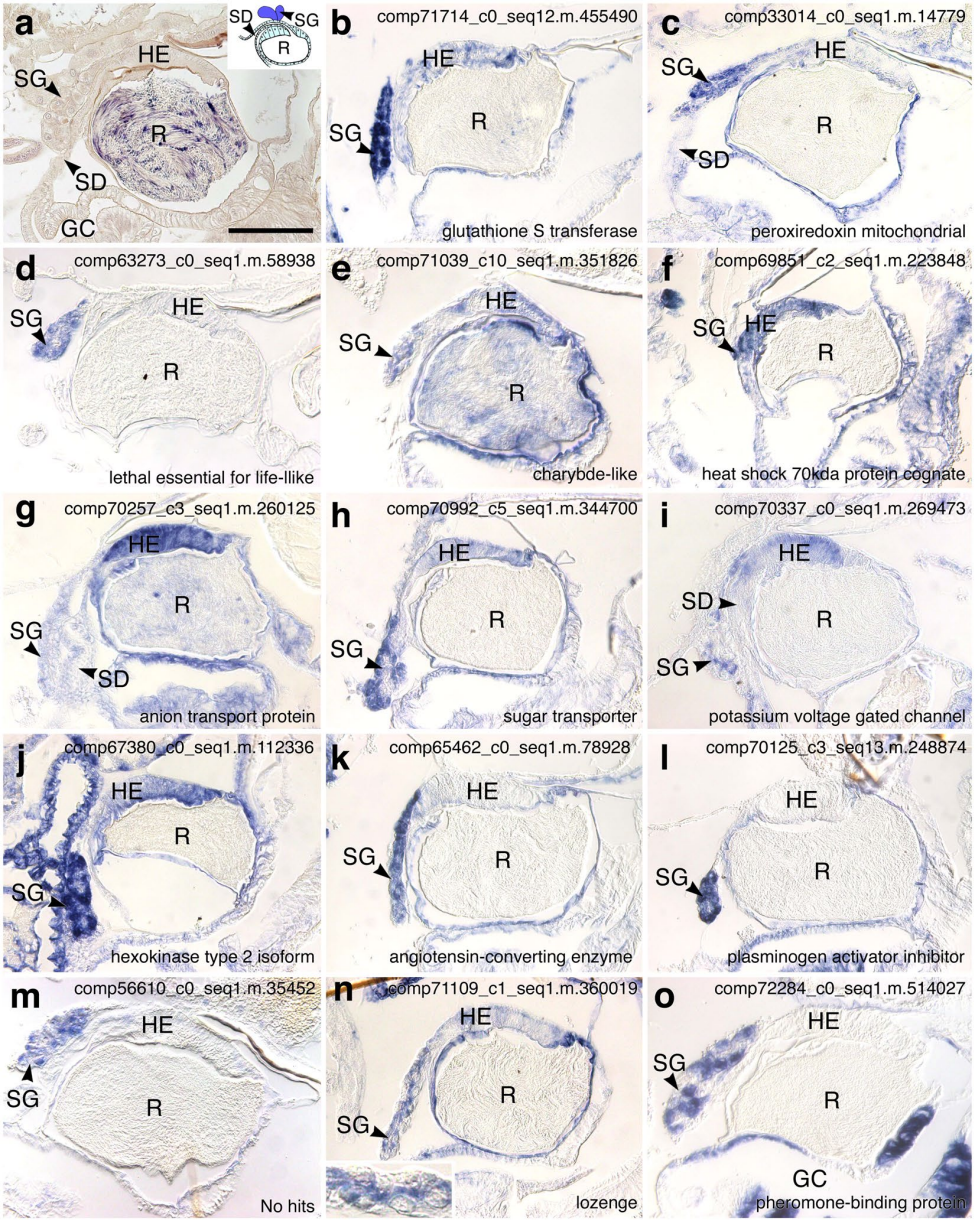
Longitudinal section of the spermatheca stained with hematoxylin and eosin (**a**) and expression patterns of selected highly expressed contigs in the spermatheca (**b**–**o**). Schematic indications of spermatheca morphology are shown in the upper right corner (**a**). Details of the spermathecal gland are shown in the bottom left corner (**n**). Scale bar, 100 µm; GC, genital chamber; HE, hilar columnar epithelium of the spermathecal reservoir; R, reservoir; SD, spermathecal duct; SG, spermathecal gland.

**Figure 6:**
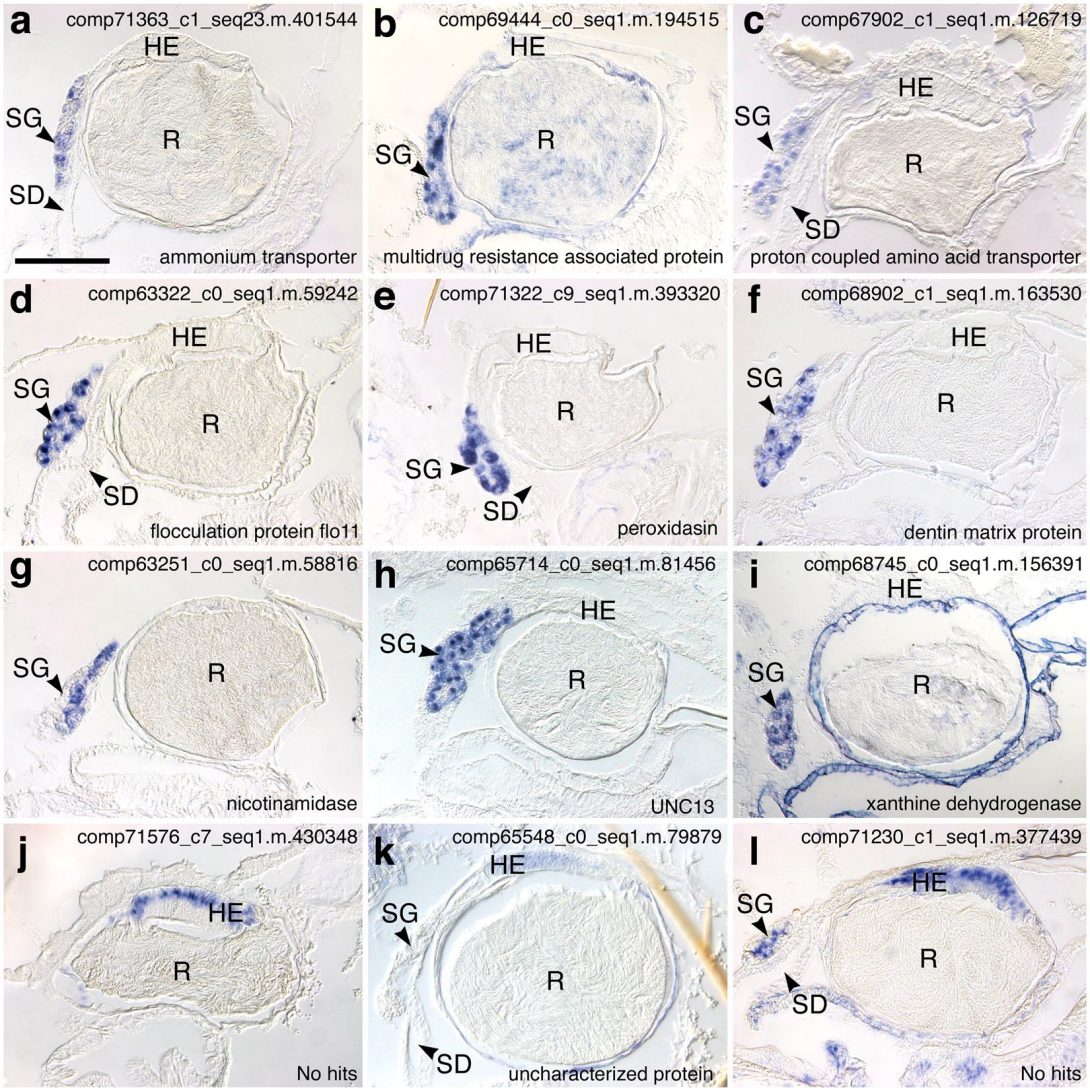
Expression patterns of 12 contigs that were only expressed in the spermatheca. Scale bar, 100 µm; HE, hilar columnar epithelium of the spermathecal reservoir; R, reservoir; SD, spermathecal duct; SG, spermathecal gland.

Although signals were detected for 117 of the 128 contigs, *in situ* hybridization analyses failed in some steps for 11 contigs. However, 31 of the remaining genes gave insignificant signals in the spermatheca (Supplementary Dataset 1), and genes for 86 contigs were detected at least in a part of the spermatheca, the hilar columnar epithelium of the spermathecal reservoir, the spermatheca duct, and secretory and duct cells of the spermathecal gland (Figs 5 and 6, Table 1 and Supplementary Dataset 1).

### Spermatheca-specific genes

Although most examined genes were expressed in the spermatheca and in other abdominal tissues and organs, such as the ovary and midgut (Fig. 5 and Table 1), 12 contigs were specifically expressed in the spermatheca (Fig. 6 and Table 1). Among them, nine, two, and one contigs were expressed in the spermathecal glands, in the hilar columnar epithelia of spermathecal reservoirs, and in the both regions, respectively. Moreover, these spermathecal gland-specific contigs included three that were categorized into the transporter functions of ammonium transporter rh type A isoform (comp71363_c1_seq23.m.401544, Fig. 6a), multidrug resistance-associated protein 4-like isoform (comp69444_c0_seq1.m.194515, Fig. 6b), which is a member of ATP-binding cassette transporter family^44, 45^, and proton-coupled amino acid transporter 1-like isoform (comp67902_c1_seq1.m.126719, Fig. 6c). Three other contigs were associated with extracellular matrix-related proteins, including flocculation protein flo11-like isoform (comp63322_c0_seq1.m.59242, Fig. 6d), which is identical to the mucin-like protein MUC1 and is involved in intercellular adhesion in yeast^46, 47^, peroxidasin (comp71322_c9_seq1.m.393320, Fig. 6e), and dentin matrix protein 4-like protein (comp68902_c1_seq1.m.163530, Fig. 6f), which is a secretory calcium-binding protein^48^. The other three contigs corresponded with pyrazinamidase nicotinamidase (comp63251_c0_seq1.m.58816, Fig. 6g), the UNC-13 homolog d isoform (comp65714_c0_seq1.m.81456, Fig. 6h), which is essential for synaptic vesicle exocytosis during neurotransmission in *Drosophila melanogaster* and *Caenorhabditis elegans* ^49, 50^, and xanthine dehydrogenase (comp68745_c0_seq1.m.156391, Fig. 6i), which catalyses the conversion of xanthine to the strong antioxidant uric acid. Finally, coting with no sequence similarities in the NCBI database (comp71576_c7_seq1.m.430348, Fig. 6j) and contig that matched to the currently uncharacterized proteins that contain zona pellucida-like domains (comp65548_c0_seq1.m.79879, Fig. 6k) were expressed only in hilar columnar epithelia of the reservoir, and the contig comp71230_c1_seq1.m.377439 (no sequence similarities in the NCBI database, Fig. 6l) demonstrated signals in the central duct of the spermathecal gland and reservoir epithelial cells.

## Discussion

This study is the first report on large-scale gene expression profiling of the spermatheca to understand the molecular mechanisms of long-term sperm storage in ant queens. Initially, we identified genes that are expressed at higher levels in the spermatheca than in the body samples and performed GO enrichment analyses of these crucial candidates for sperm storage functions. GO terms that are related to energy production were found to be enriched among genes that are highly expressed in the spermatheca, suggesting high energy costs of spermatheca function in ant queens.

We also performed two sets of differential gene expression analyses of the spermatheca with and without sperm and at 1 week and 1 year after mating to aim screening of enhanced genes triggered by insemination and by prolonged sperm storage. In these analyses, differential expression of genes of the GO category oxidoreductase activity was pronounced, suggesting the requirement of enhanced antioxidant functions. Antioxidant enzymes function as scavengers of free radicals. The production and elimination of reactive oxygen species (ROS) was reportedly associated with sperm longevity in humans^51, 52^. GO enrichment analyses also suggested that antioxidant function is enhanced in the spermatheca at 1 week after mating compared with the virgin spermatheca and the spermatheca at 1 year after mating, indicating that antioxidant functions of the spermatheca were enhanced in the initial phase rather than in the later phase after mating. Hence, ROS levels in sperm cells may be higher soon after ejaculation by males, but the spermatheca remove ROS in the initial phase after sperm transmission, subsequently the spermatheca may maintain stored spermatozoa with lower resource for antioxidant function after establishment of suitable sperm conditions in the later phase.

Although all genes that were differentially expressed in the spermatheca samples are likely candidates for further studies of spermatheca functions, we focused on spermatheca-enriched genes with elevated expression following mating (inseminated vs. virgin queens) and following long duration of sperm storage (at 1 week vs. at 1 year after mating). Although log fold changes in spermatheca gene expression levels in inseminated queens were less than 1 compared with those in virgin queens, we identified only one contig hit for the pheromone-binding protein gp-9-like (comp72284_c0_seq1.m.514027). This gene was strongly expressed in the spermathecal glands and in the genital chamber (see below), suggesting important roles in reproductive functions of ant queens. This contig was also associated with a soluble odorant-binding protein with homology to proteins that were identified in seminal fluids and accessory glands of other insect males, such as fruit fly, mosquitos, crickets, and flour beetles^53–56^, however the function in male reproductive tracts remain unclear. In contrast with these short-term sperm storage species, the corresponding gene was expressed at significantly lower levels in *C*. *osakensis* male accessory glands than in their body tissues (Gotoh *et al*., in prep.). In honeybees, odorant-binding protein 14 has also been detected in spermathecal fluid of queens, but not in seminal fluid of males^12, 13^, as well as *C*. *osakensis*. These data suggest that the reproductive functions of this gene may differ between long-term sperm storage species, honeybee and *C*. *osakensis*, and other short-term sperm storage species, and are likely to have central roles of long-term sperm maintenance, although we cannot discard the possibility that the differences reflect phylogenetical difference between hymenopteran and non-hymenopteran species.

Based on the present comparisons of differentially expressed genes, we selected 128 contigs potentially involved in the prolonged sperm storage and investigated their expression patterns in various spermatheca parts, including the hilar columnar epithelium of the spermathecal reservoir, the spermatheca duct, and secretory and duct cells of the spermathecal gland. In previous studies on ant and honeybee queens, the function of the spermathecal gland and the hilar columnar epithelium of the spermathecal reservoir were considered important for sperm maintenance. Moreover, proteins from spermathecal glands in honeybees were reportedly secreted into the spermathecal reservoir to enhance sperm viability^30^. Hence, the genes expressed in the spermathecal gland and containing signal sequences may encode proteins for secretion into the spermathecal reservoir, leading to direct effects on sperm longevity in ants. Specifically, proteases and protease inhibitors containing signal sequences were expressed in the spermathecal glands of ant queens, likely influencing sperm physiology after secretion into the spermathecal reservoir. Serine protease genes with signal sequences were reportedly dominant in spermatheca from various *Drosophila* species, and the encoded proteins were suggested to be secreted into the spermathecal lumen^57, 58^. Although the functions of these proteins have not been characterized in insect females, the serine protease trypsin induced sperm motility and maturation in males of Lepidoptera species and water striders^59–62^. Moreover, proteases and related proteins have been associated with sperm maturation and fertilization in mammals^63^.

Previous ultrastructural observations in honeybees and ants indicated ion-transporting functions of the columnar epithelium of the spermathecal reservoir, but these cells showed no secretory functions^20, 24^. In agreement, we identified contigs hit for transporters and channels that likely regulate chemical components, such as sugars, ions, and amino acids, in the spermathecal reservoir and they may affect microenvironments surrounding sperm. However, these assertions require confirmation of chemical components in spermathecal fluids from ant queens. Although functions of the columnar epithelium have not been suggested except for transporting roles in previous studies^20, 24^, our analyses of spatial expression patterns using *in situ* hybridization detected genes from various functional categories, including antioxidant enzymes, molecular chaperones, metabolic pathways, and extracellular matrix-related proteins, and some contigs with no sequence similarities in the NCBI database. Hence, the columnar epithelium may have multiple functions, and further analyses of the present differentially expressed genes will provide new insights into spermatheca functions and mechanisms of long-term sperm storage in ant queens.

Genes that were matched to antioxidant enzymes and molecular chaperones were highly expressed in the hilar columnar epithelium of the spermathecal reservoir and/or the spermathecal gland. Protein chaperones have been shown to prevent protein aggregation and misfolding^64^, and among these, various heat shock proteins have been associated with sperm functions, spermatogenesis, sperm maturation, and sperm-egg interactions during fertilization in mammals^65^. In addition, antioxidant enzymes and molecular chaperones are reportedly expressed in honeybee spermatheca^10, 12, 66^, suggesting conserved contributions to long-term sperm storage. However, it remains unclear whether these molecules directly affect sperm cells or are associated with spermatheca maintenance independently of sperm.

In the present study, we showed spermatheca-specific expression of 12 contigs. To our knowledge, these genes have never been reported in female reproductive organs of animals and were not overexpressed in sperm storage organs of female *Drosophila* species^57, 58^, indicating specialized roles in ant spermatheca and suggesting associations with long-term sperm storage mechanisms. In our *in situ* hybridization analyses, three of nine spermathecal gland-specific contigs were matched to extracellular matrix-related genes. In humans, semenogelin is a major component of seminal fluid that contributes to gel matrix formation^67^ and reportedly inhibits sperm mortality through the formation of physical coagulum traps^68^. In contrast with insect species with short-term sperm storage, ant queens immobilize sperm cells within the spermatheca, suggesting that sperm immobilization contributes to sperm survival (Gotoh *et al*. in prep.). Hence, because the spermathecal fluid is viscous (Gotoh pers. obs.), these extracellular matrix-related proteins may be secreted into the lumen of the spermathecal reservoir to influence viscosity and sperm motility.

Long-term sperm storage is a hallmark of social Hymenoptera and *C*. *osakensis* queen is a good model for understanding the mechanisms on the very long-term sperm storage. Our study will provide an important resource for future studies on molecular and cellular mechanisms of the prolonged sperm storage in *C. osakensis* and the evolution of the advanced society depending on the prominent reproductive ability in social Hymenoptera.

## Methods

### Sample collection

*C. osakensis* queens and males were collected during nuptial flight in the Kagawa and Aichi prefectures, Japan. Dealated queens were reared at room temperature for 1 week or 1 year after nuptial flight and the spermathecae from queens and the accessory glands from males were dissected in 1 × phosphate buffered saline (PBS). The mating status of queens was confirmed during dissection. Worker ants were obtained from a rearing colony (Supplementary Table S1).

### RNA extraction and library preparation

Total RNA was isolated from the bodies and 7–20 spermathecae (spermathecal reservoir, the spermathecal gland, the sperm pump, and a part of the spermathecal duct) of queens with different ages and reproductive statuses using RNeasy kits (Qiagen) according to manufacturer’s instructions (Supplementary Table S1). Total RNA was also isolated from 6–10 male bodies using the TRIzol reagent (Invitrogen) and from 10–20 male accessory glands and from five worker bodies of different ages using RNeasy kits (Qiagen) according to the manufacturer’s instructions (Supplementary Table S1). Queen and male RNA samples were prepared in triplicate. Due to the difficulty of queen sample preparation, two of the three queens’ body samples lacked abdominal tips (Supplementary Table S1). Sequence libraries were generated using TruSeq RNA sample preparation kits v2 (Illumina) following manufacturer’s protocol with minor modifications: RNA fragmentation was conducted for 4 min instead of 8 min at 94 °C and the number of PCR cycles was changed from 15 to 10 for all but spermatheca samples, which had low RNA concentrations. Gel size selection was conducted to remove fragments of more or less than 200–500 base pairs from the four spermatheca libraries (Supplementary Table S1). We validated the sequence libraries using qPCR (KAPA SYBR FAST qPCR kit, Kapa Biosystems, Woburn, MA USA) and Bioanalyzer High Sensitivity DNA Assay (Agilent Technologies).

### Sequencing and data analysis

Paired end sequencing of the 23 libraries was performed in two lanes of a HiSeq2000 flow cell. Qualities of sequences were assessed using a FastQC program (http://www.bioinformatics.bbsrc.ac.uk/projects/fastqc/). *De novo* assembly of short reads was performed using Trinity^42^ (v. r2012-06-08). Open reading frames (ORFs) were predicted using the TransDecoder program (Trinity package). Short reads were mapped to the reference gene set using Bowtie2 (v. 2.1.0) and the transcript abundances were estimated using eXpress (v. 1.4.1). Sequence similarity search was performed against the NCBI’s non-redundant (nr) protein database (ver. October 2015) using BLASTP (e-value cut-off of 1.0e-4). Based on the BLAST nr search result, we removed contaminant contigs with bacterial sequence hits. We also excluded very lowly expressed contigs with reads per kilobase of exon per million mapped reads (RPKM) values of less than 1.0 in all 23 samples. Secretion signal sequences and protein domains were predicted using SignalP (v. 4.0) and Pfam databases (v. 27.0, e-value cut-off of 1e-6), respectively. Gene Ontology (GO) terms were assigned using Blast2GO software (v. 3.3.5). GO term enrichment analyses were performed using Blast2GO to test over-representation by comparing differentially expressed genes with all annotated genes. Differentially expressed genes analyses were conducted using DEseq2^43^.

### Paraffin sectioning and RNA *in situ* hybridization

Specific primers for 128 contigs, including 148 ORFs, were designed to amplify 600–1000-base pair lengths of probe fragments (Supplementary Dataset 1). For seven contigs including 19 ORFs, probes were designed for untranslated regions (UTR; Supplementary Dataset 1). After cloning into the pTA2 vector (TOYOBO), sense and antisense probes were labelled with digoxigenin-UTP (Roche) using T7 or T3 RNA transcription kits (Roche) and concentrations were adjusted to 100 ng/µl in formamide. For contigs that lacked coding sequence information, sense or antisense orientations were assumed from corresponding signal data.

Abdomens were dissected from queens collected in Aichi and Hyogo prefectures at 1 week and 1 year after mating and were fixed in 4% paraformaldehyde in phosphate buffered saline for 2 h. We used 1 week or 1 year samples for *in situ* hybridization according to the higher expression of selected genes. Tissues were dehydrated in a graded ethanol series and were replaced with butanol before embedding in paraffin. Longitudinal serial sections were cut at a thickness of 9 µm, were deparaffinised in xylene, and then were dehydrated in an ethanol series. After washing, sections were incubated with 10-µg/ml proteinase K (Promega) at 37 °C for 15 min and were then refixed in 4% paraformaldehyde. Acetylation was performed for 10 min using 100-mM triethanol amine and 0.25% acetic acid anhydride. Subsequently, prehybridization solution was replaced with 400-ng/ml probe diluted in hybridization solution and was incubated overnight at 51 °C. Sections were then washed and incubated in a 1:1000 dilution of anti-digoxigenin (Roche) for 2 h at room temperature and were then washed again. After washing in solution containing 5-mM MgCl_2_, 100-mM NaCl, 100-mM Tris (pH 9.5), and 0.1% Tween-20, tissues were exposed to NBT/BCIP solution (Roche) until signals were detected. For histological observation of the spermatheca, longitudinal serial sections of the abdomen were cut at 4 µm, and stained with hematoxylin and eosin as previously described^69^. Specimens were observed (Leica DMRB and Olympus BX53) and were photographed with a 3CCD digital camera (Victor KY-F75 and Olympus DP72).

## Electronic supplementary material

Supplementary Table S1

Dataset 1
