# Supplementary material for: Transcriptome profiling of the spermatheca identifies genes potentially involved in the long-term sperm storage of ant queens
Source: Sci Rep. 2017 Jul 20;7:5972. doi: 10.1038/s41598-017-05818-8 (PMC5519678; doi:10.1038/s41598-017-05818-8)
Supplement: Supplementary file 1 — Supplementary Table S1 [file 41598_2017_5818_MOESM1_ESM.pdf]

Supplementary information

Transcriptome profiling of the spermatheca identifies genes potentially involved in the long-term sperm storage of ant queens

Ayako Gotoh[1], Shuji Shigenobu[2,3], Katsushi Yamaguchi[2], Satoru Kobayashi[4], Fuminori Ito[5] and Kazuki Tsuji[6]

[1] Department of Biology, Faculty of Science and Engineering and Institute for Integrative Neurobiology, Konan University, 8-9-1 Okamoto, Higashinada-ku, Kobe 658-8501, Japan

[2] NIBB Core Research Facilities, National Institute for Basic Biology, Okazaki 444-8585, Japan

[3] Department of Basic Biology, Faculty of Life Science, SOKENDAI (The Graduate University for Advanced Studies), Okazaki, Aichi 444-8585, Japan

[4] Life Science Center of Tsukuba Advanced Research Alliance (TARA Center), University of Tsukuba, Tsukuba, Ibaraki 305-8577, Japan

[5] Faculty of Agriculture, Kagawa University, Ikenobe, Miki, 761-0795, Japan

[6] Department of Agro-Environmental Sciences, Faculty of Agriculture, University of the Ryukyus, Nishihara, Okinawa, 903-0213, Japan

Supplementary Table S1 Information of samples and preparation methods for sequencing libraries.

Sample No.	Sample name	Collection year and site	No. of individuals	State of body samples	RNA extraction method	PCR cycles of cDNA	Gel extraction
1	Spermatheca 1week unmated	2011 in Aichi pref.	20	-	RNeasy kit	10	No
2	Spermatheca 1week unmated	2011 in Aichi pref.	10	-	RNeasy kit	15	No
3	Spermatheca 1week unmated	2011 in Aichi pref.	10	-	RNeasy kit	15	Yes
4	Spermatheca 1week mated	2011 in Aichi pref.	20	-	RNeasy kit	10	No
5	Spermatheca 1week mated	2011 in Aichi pref.	8	-	RNeasy kit	15	Yes
6	Spermatheca 1week mated	2011 in Aichi pref.	9	-	RNeasy kit	15	No
7	Spermatheca 1year mated	2010 in Kagawa pref.	19	-	RNeasy kit	10	No
8	Spermatheca 1year mated	2010 in Kagawa pref.	7	-	RNeasy kit	15	Yes
9	Spermatheca 1year mated	2010 in Kagawa pref.	8	-	RNeasy kit	15	Yes
10	Queen body 1week mated	2011 in Aichi pref.	1	Whole	RNeasy kit	10	No
11	Queen body 1week mated	2011 in Aichi pref.	1	Dissected	RNeasy kit	10	No
12	Queen body 1week mated	2011 in Aichi pref.	1	Dissected	RNeasy kit	10	No
13	Queen body 1year mated	2010 in Kagawa pref.	1	whole	RNeasy kit	10	No
14	Queen body 1year mated	2010 in Kagawa pref.	1	Dissected	RNeasy kit	10	No
15	Queen body 1year mated	2010 in Kagawa pref.	1	Dissected	RNeasy kit	10	No
16	Male body	2011 in Aichi pref.	7	Whole	Trizol	10	No
17	Male body	2011 in Aichi pref.	6	Whole	Trizol	10	No
18	Male body	2011 in Aichi pref.	10	Dissected	Trizol	10	No
19	Accessory gland	2011 in Aichi pref.	20	-	RNeasy kit	10	No
20	Accessory gland	2011 in Aichi pref.	10	-	RNeasy kit	10	No
21	Accessory gland	2011 in Aichi pref.	10	-	RNeasy kit	10	No
22	Worker body	2008 in Kagawa pref.	5	Whole	RNeasy kit	10	No
23	Worker body	2008 in Kagawa pref.	5	Whole	RNeasy kit	10	No
